# Expression, Purification, Structural and Functional Characterization of Recombinant Human Parvulin 17

**DOI:** 10.1007/s12033-022-00493-1

**Published:** 2022-04-25

**Authors:** Alessandra Monti, Raffaele Ronca, Giuseppe Campiani, Menotti Ruvo, Nunzianna Doti

**Affiliations:** 1 Instituto di Biostrutture e Bioimmagini-CNR, Via Pietro Castellino 111, 80131 Napoli, Italy; 2 Dipartimento di Biotecnologie, Chimica e Farmacia, Via Aldo Moro, 2, 53100 Siena, Italy

**Keywords:** Parvulins, Par17, Pin1, Circular Dichroism spectroscopy, Size-Exclusion Chromatography, FRET-based peptidyl-prolyl *cis–trans* isomerase assay, High Throughput Screening (HTS)

## Abstract

Parvulins, peptidyl-prolyl isomerase enzymes (PPIase), catalyze the *cis–trans* isomerization of prolyl bonds in polypeptides, contributing to folding and function regulation of many proteins. Among Parvulins, Par17, exclusively expressed in hominids, is the least examined in terms of structure, catalytic function and cellular activity. Setting the conditions for the preparation of recombinant active Par17 may therefore significantly foster future studies. Here, we comparatively evaluated the impact of several parameters, including host strains, culture media, isopropyl ß-D-1-thiogalactopyranoside concentration, post-induction incubation time and temperature, on the overexpression of Par17 in *E. coli* cells. A similar approach was also comparatively adopted for the preparation of the recombinant full-length Pin1 protein, the most representative Parvulin, and the catalytic domains of both enzymes. Proteins were efficiently expressed and purified to homogeneity and were subjected to a structural characterization by Size Exclusion Chromatography and Circular Dichroism. Moreover, a single-step homogeneous protease-based fluorimetric assay, potentially scalable in HTS format, has been developed for determining the peptidyl-prolyl *cis–trans* isomerase activity of recombinant Parvulins. Results obtained show that proteins are folded and active. These new data mark an important milestone for progressing the investigation of Parvulins.

**Supplementary Information:**

The online version contains supplementary material available at 10.1007/s12033-022-00493-1.

## Introduction

Peptidyl-prolyl *cis–trans* isomerases (PPIases) catalyze the isomerization of prolyl bonds in peptides and proteins regulating their folding, subcellular localization, stability, activation, and interaction with multiple other proteins [1, 2]. The PPIase superfamily includes cyclophilins, FK506-binding proteins (FKBPs) and parvulins [1, 2].

The name Parvulins, from the Latin: “parvulus”, which means very small (~ 10 ÷ 17 kDa) [3], is related to their low molecular weight as compared to other isomerases [3].

The human genome contains two parvulin genes: *pin1* and *pin4*. The most representative and the best characterized human Parvulin is Pin1. It catalyzes the *cis–trans* isomerization of amide bonds between phosphorylated serine or threonine and proline (pSer/pThr-Pro) within polypeptide chains [1, 2]. The conformational changes catalyzed by Pin1 after protein phosphorylation regulate the function of key proteins involved in several physiological and pathological processes, including β-catenin, tau, APP, and Notch1 in neurons [4–12].

*Pin4* gene encodes two proteins via alternative transcription initiation: Par14 (13.8 kDa; 131 aa) and Par17 (16.6 kDa; 156 aa) [13]. Noteworthy, Par14 has found in all multicellular organisms whereas Par17 is present exclusively in great apes and humans [14]. Par14, mainly located in the nucleus, is able to bind the DNA and has been associated with chromatin remodeling, cell cycle progression, rRNA processing, tubulin polymerization and tumor growth [13–24]. However, while the signaling pathways involving Pin1 have been extensively studied, the role of Par14 is not yet fully elucidated. Very little is known about Par17. Par14 and Par17 differ only in their N-terminal extensions (Fig. S1), indeed an additional fragment of 25 residues, uniquely presents in Hominidae [14, 25], is preceding the N-terminal extension in Par17. Although Par17, like Par14, is able to bind DNA in vitro, it is located mainly in the cytoplasm, especially in the mitochondrial matrix or colocalized with microtubules [12, 16]. Recently, it has been shown that in vitro Par17 catalyzes tubulin polymerization 2.5-fold more efficiently than Par14 [23], suggesting that the N-terminal region of Par17 plays a critical role in this process. Moreover, it has been demonstrated that, like Par14, Par17 is involved in hepatitis B virus (HBV) replication, highlighting a role for Par17 as a new therapeutic target for controlling HBV infection [19].

From a structural point of view, human Pin1 comprises an N-terminal WW domain and a C-terminal PPIase domain, whereas Par14 and Par17 both lack the WW domain [26]. The catalytic domains of three proteins show a high degree of structural conservation [22, 27]. The global fold of the PPIase domain of Par14/Par17, which comprises three α-helices and a four-stranded β-sheet, is indeed superimposable with that of the catalytic domain of Pin1, though that of Pin1 is considerably longer (114 residues compared to 96 residues of both Par14 and Par17; see Fig. S1) and shares only 30% sequence identity with the other two (Fig. S1). The N-terminal fragment of Par14 is unstructured [20, 22], whereas the N-terminal extension of Par17 was predicted to be an amphypatic helix [25] and supposedly functions as a novel mitochondrial targeting signal [14]. However, apparently the N-terminal region of Par17 features a mostly random-coil conformation in solution [28]. Considering the importance of the N-terminal elongation for Par17 function and localization [14, 23, 25], it was assumed that this region may adopt a functionally-relevant conformation only in the presence of physiological ligands but remains largely unstructured in the isolated form [28].

Here, with the aim to foster further research on Par17, an overlooked drug target which might also provide a deeper understanding of human-specific evolution, we have developed a protocol for the easy and fast production of a folded and active recombinant protein.

In fact, whereas Pin1 has been efficiently expressed already by several groups [29, 30], the preparation of Par17 has been so far described only in very few reports [23, 28] fused with the GST protein (MW = 27 kDa). The purification protocol included the extraction of GST-fused Par17 using GST-immobilized supports, removal of GST with proteases and other subsequent purification steps, making the process time-consuming and expensive.

Here, we have overexpressed Par17 with a short tag at the N-terminus (His6-tag) in *E. coli* cells. The purified recombinant protein has been characterised structurally and functionally. Moreover, to perform a full structural and functional comparison between Par17 and Pin1, we have also expressed and purified both full-length proteins as His6-fused polypeptides and the corresponding catalytic domains without tags. Protein structure and oligomeric states in solution have been evaluated by size exclusion chromatography (SEC) and circular dichroism (CD), while their isomerase activity has been assessed by a new validated, ad hoc designed, single-step homogeneous protease-based fluorimetric assay, which is suitable for all Parvulins and potentially scalable in HTS modality.

## Material and Methods

### Substrates and Chemicals

Expression plasmids were purchased from GeneScript (Netherlands). The HRP conjugated anti-His monoclonal antibody (mouse) and the anti-GST monoclonal antibody (rabbit) were from BioRad (Milan). GSTrap, HisTrapHP, Superdex 75 and 200 10/300 columns were from GE Healthcare (Milan). Bacterial expression strains and the cloning strain TOP10 were from Invitrogen (Milan). The protein molecular marker was purchased from Bio-Rad (Milan). 9-fluorenylmethoxycarbonyl (Fmoc) protected amino acids (purity > 99%), 1-[Bis (dimethylamino)-methylene]-1H-1,2,3-triazole-[4,5-b] pyridinium-3-oxide hexafluorophosphate (HATU), ethyl-2-cyano-2-(hydroximino) acetate (Oxyma), N,N'-diisopropylcarbodiimide (DIC), triisopropylsilane, piperidine, 2,4,6-trimethylpyridine (sym-collidine), N,N-diisopropylethylamine (DIPEA), α-Chymotrypsin (hereafter only Chymotrypsin) from bovine pancreas (TLCK-treated to inactivate residual trypsin activity), dimethylformamide (DMF), methanol (MeOH), trifluoroacetic acid (TFA), diethyl ether (Et_2_O) and all solvents used for HPLC were provided by Merck (Darmstadt, DE). Fmoc-Glu(EDANS)-OH and Fmoc-Lys(Dabcyl)-OH were from PolyPeptide Group (Strabourg, France). The HPLC columns were purchased from Phenomenex (Torrance, CA, USA).

### Expression of Full-Length Proteins and Catalytic Domains in E. coli Cells

The constructs of full-length proteins and of their corresponding catalytic domains (see Fig. S1) (Pin1-pET14b, Par17-pET14b, Pin1(50–163)-pGEX4T1 and Par17(60–156)-pGEX4T1) were transfected in *E. coli* cells. Several expression conditions including cell media (LB and SOC), strains of E. coli (BL21(DE3) and BL21(DE3)pLysS), culture temperature (37 °C and 22 °C), incubation time (3 and 16 h) and IPTG concentration (0.3, 0.5 and 1.0 mM) were tested to optimize the overexpression of the target proteins. In all cases, 100 µl of the overnight culture was inoculated in 10 ml of pre-warmed medium containing 50 μg/ml of ampicillin at 37 °C under 180 rpm shaking. Once achieved the optimal optical density at 600 nm (OD_600_) between 0.6 and 0.8, the overexpression was induced with IPTG. Subsequently the cells were harvested by centrifugation (20 min, 4 °C, 6000 rpm), resuspended in lysis buffer (20 mM Tris–HCl, 500 mM NaCl, 1 mM DTT; pH 7.5) containing protease inhibitor mixture (Complete Protease Inhibitor Tablets, Roche) and then sonicated. Next, 15 μl of supernatant from each sample, added with 5 μl of 4X SDS-PAGE sample buffer (0.25 M Tris–HCl pH 6.8 0.5 M DTT 10% SDS 50% Glycerol 0.5% bromphenol blue) was boiled at 90 °C for 3 min, centrifuged for 1 min at 14,000 rpm and then loaded on 15% (full-length parvulins and GST-fused proteins) or 4–20% (precast) SDS-PAGE gels (catalytic domains). Gels were electrophoresed at 200 V for 1 h and finally stained with Comassie blue R-350 (0.1% (w/v) Coomassie blue R-350, 20% (v/v) methanol, and 10% (v/v) acetic acid).

### Large-Scale Production of Recombinant Proteins

Large-scale production of Parvulins was performed under optimized conditions (see Table S1). Pre-culture was made in 5 ml of the selected medium containing 50 μg/ml ampicillin inoculated with 50 μl of the specific glycerol stock. Subsequently, 600 ml of medium/antibiotic was inoculated with 5 ml of pre-cultured media.

### Purification of Recombinant Protein

Par17 and Pin1 were overexpressed as N-terminally His6-tagged proteins (hereafter, Par17 and Pin1, respectively), whereas the catalytic domains of both proteins, spanning residues 60–156 and 50–163 (hereafter, Par17(60–156) and Pin1(50–163)) were overexpressed as N-terminally GST-fused proteins (see Fig. S1). Once defrosted, pellets previously resuspended in the lysis buffer were left at room temperature under gentle stirring for 30 min and then sonicated for 20 min (10 s on and 10 s off) in ice. Subsequently, samples were centrifugated at 4 °C for 15 min at 16,000 rpm and soluble fractions were subjected to purification steps. In particular, for the 6His-tagged full-length proteins, the soluble fractions were loaded onto a HisTrapHP affinity column (1 ml) at a flow rate of 0.5 ml/min using an AKTA purifier system (GE Healthcare BioScience AB, Uppsala, Sweden). The column was washed with 10 volumes of washing buffer (50 mM Tris–HCl, 150 mM NaCl, 0.1 mM DTT; pH 8.0). Proteins were eluted at a flow rate of 0.5 ml/min using an imidazole step gradient (imidazole 100, 300 and 500 mM). 15 µl of each collected fraction were analysed by 15% SDS-PAGE and western blot (WB) using the HRP conjugated anti-His antibody. Fractions of interest were dialyzed using 3.5 kDa membranes (Thermo Scientific) against 50 mM Tris–HCl pH 8.5, 150 mM NaCl for 16 h at 4 °C.

The soluble fractions of bacterial lysates containing the GST-tagged catalytic domains were loaded onto a GSTrap affinity column (1 ml) at a flow rate of 0.2 ml/min using an AKTA purifier system (GE Healthcare BioScience AB, Uppsala, Sweden). After the washing step, proteins were eluted with 10 mM reduced glutathione (GSH). The fractions containing the domains were analyzed by 4–20% precast SDS-PAGE gels and WB. 5 mg of each GST-protein in a total volume of 10 ml (0.5 mg/ml), were subjected to cleavage with thrombin (one unit for every 100 µg of protein) in a dialysis bag at 22 °C for 16 h in Tris–HCl 50 mM pH 8.5, 150 mM NaCl buffer. The dialyzed samples were then loaded again on the GSTrap column under the conditions reported above to remove the GST. Pure products were analysed on SDS-PAGE gels at 15% and 4–20% for the GST-tagged proteins and the catalytic domains, respectively.

Protein concentration was determined spectrophotometrically at 280 nm with a NanoDrop 2000c UV–Vis spectrophotometer (Thermo Scientific) using for each protein the theoretical molecular weight and the molar extinction coefficient ε, calculated using the Expasy tools (ProtParam™, web.expasy.org/protparam) provided by the Swiss Institute of Bioinformatic, ε_280nm_ Par17: 8480 M^–1^ cm^–1^; ε_280nm_ Pin1: 21,095 M^–1^ cm^–1^; ε_280nm_ Par17(60–156): 8480 M^–1^ cm^–1^; ε_280nm_ Pin1(50–163): 7115 M^–1^ cm^–1^. The yield of purified proteins in mg per liter of *E. coli* culture was determined from the absorbance at 280 nm after the purification step.

### Size Exclusion Chromatography Analyses

The oligomeric state of the recombinant proteins was evaluated by a semi-preparative size-exclusion chromatography column (SEC) (Superdex 75 10/300, GE Healthcare) using a calibration curve obtained with the following standards: blue dextran (MW: 2,000,000), bovine serum albumin (MW: 66,399 Da), ovalbumin (MW: 44,000 Da), carbonic anhydrase (MW: 29,000 Da) and cytochrome c (MW: 12,400 Da). The operating conditions were selected on the basis of the theoretical size (MW) and isoelectric point (IP) of target proteins calculated by Compute pI/MW (https://web.expasy.org/compute_pi/) (pI/MW: 8.95/20406.74 for Pin1; pI/Mw: 10.04 / 18634.48 for Par17; pI/Mw: 8.01 / 13174.79 for Pin1(50–163); pI/Mw: 9.37 / 11287.13 for Par17(60–153)). Proteins were loaded on SEC column at 0.5 ml/min in Tris–HCl 50 mM pH 8.5, 150 mM NaCl buffer.

### Circular Dichroism Spectroscopy and Thermal Denaturation Studies

Far-UV circular dichroism spectra of recombinant parvulins were recorded using a Jasco J-715 spectropolarimeter, equipped with a PTC-423S/15 Peltier temperature controller, in a 0.1 cm quartz cell. All spectra were acquired according to the following parameters: far UV range between 190 and 260 nm, band width: 1 nm, response: 8 s, data pitch: 0.2 nm, scanning speed: 10 nm/min. Spectra were collected at 25 °C at a protein concentration of 10 μM in 5 mM phosphate buffer at pH 7.4. Three spectra were accumulated and averaged to obtain final spectra.

The thermal unfolding of parvulins were measured at a fixed 222 nm wavelength during heating from 25 to 95 °C. CD intensity was expressed as mean residue ellipticity (deg × cm2 × dmol − 1 of residue) calculated referring to the total amino acid content.

### Chemical Synthesis and Purification of Substrates

Two distinct peptide substrates were designed and prepared for the FRET- and HPLC-based analyses, respectively. The sequences are reported in the section of Results. All peptides were synthesized on solid phase following the Fmoc (N-9-Fluorenylmethyloxycarbonyl) strategy and following an optimized protocol reported in literature [31]. The FRET substrate was acetylated at the N-terminus by treatment with a solution of acetic anhydride 30% and DIPEA 5% in DMF for 30 min. On the peptide substrate for the HPLC-based analysis the Fmoc group was left at the N-terminus to favor reverse phase retention and identification of products by the characteristic UV absorption spectrum. After cleavage from the resins and lyophilization peptides were purified by RP-HPLC on a WATERS 2545 preparative system (Waters, Milan) equipped with a WATERS 2489 UV/Vis detector set at at 214 nm and using a Nucleodur HTec C18 column (5 μm, 150 × 21 mm) operated at a flow rate of 12 ml/min. A linear gradient from 5 to 70% of solvent B (0.1% TFA in acetonitrile, ACN) in 15 min was applied to separate the peptides from impurities.

### MS Analyses

The identity of proteins and peptides was evaluated by LC–MS analyses using an ESI-TOF–MS Agilent 1290 Infinity LC System coupled to an Agilent 6230 time-of-flight (TOF) mass spectrometer (Agilent Technologies, Cernusco sul Naviglio, Italy). The LC module equipped with a binary solvent pump degasser was also coupled with a photodiode array (PDA) detector, with a column heater and with an autosampler. About 1 µg of recombinant proteins were loaded on a C4 (3 mm, 4.6 Å ~ 50 mm) column and analysed using a linear gradient starting from 10 to 70% of 0.05% TFA in ACN in 30 min at a flow rate of 0.2 ml/min. Peptides were analysed injecting 200 ng on C18 columns and applying a linear gradient starting from 10 to 70% of 0.05% TFA in ACN in 30 min at 0.2 ml/min.

### Evaluation of Parvulins Catalytic Activity by HPLC Analyses

Time- and concentration-dependent isomerase activities of recombinant parvulins were evaluated by HPLC using the Fmoc-protected substrate (see below for the complete sequence and structure). The conditions used were as follows: column Nucleodur C18 HTec (3 μm, 50 × 2 mm) equilibrated at a flow rate of 0.2 ml/min; linear gradient from 10 to 70% of 0.1% TFA in ACN in 20 min; wavelength 265 nm. The differences between Parvulins-catalyzed and spontaneous *cis–trans* isomerization rates of the substrate were evaluated by monitoring the reduction of the Fmoc-protected full-length peptide and/or the increase of chymotrypsin-hydrolyzed Fmoc-peptide at 265 nm (maximum Fmoc absorption). The assays were carried out at room temperature and in a volume of 200 μl, using the substrates at 50 ng/μL, commercial Chymotrypsin at 200 ng/μL and Parvulins at 200 ng/μL. Assays were performed in Hepes 35 mM and LiCl 5 mM buffer, pH 7.4.

### Automated FRET-Based Chymotrypsin-Coupled Prolil-Peptidyl Cis–Trans Isomerase Assay

The chymotrypsin-coupled prolil-peptidyl *cis–trans* isomerase assay was set up using a peptide similar to that previously reported for CypA [32]. The new substrate has the following structure: Ac-EK(Dabcyl)pSPRFE(EDANS)KA-NH_2_, and is therefore endowed with the FRET probes EDANS and Dabcyl [32], a widely used donor-quencher pair. The optimal absorbance and emission wavelengths of EDANS are λ_abs_ = 336 nm and λ_em_ = 490 nm, respectively, while Dabcyl has an absorption band with a maximum at 472 nm, which, to a large extent, overlaps with the emission spectrum of EDANS [32]. When the two fluorophores are in the range 10–100 Å, the energy emitted by EDANS is quenched by Dabcyl. The intact molecule is thus internally quenched, while EDANS fluorescence is readily restored upon cleavage with Chymotrypsyn on the phenylalanine and the intensity changes can be detected continuously and directly [32]. The cleavage is very slow when the proline is in *cis* conformation [32] while it is significantly accelerated in the presence of PPIases that promote the *cis–trans* conversion. The chymotrypsin-coupled enzymatic assay was performed in 384-well black solid bottom plates (Perkin Elmer) in a total volume of 50 μl in each well containing the synthetic FRET substrate, using an automated MICROLAB STAR Liquid Handling Workstation from Hamilton Robotics, following a protocol reported in literature [32]. Chymotrypsin, Parvulins and FRET substrate solutions were prepared in Hepes 35 mM pH 7.4, LiCl 5 mM buffer at a concentration fourfold that of the final concentration used in the assay. The FRET substrate at 15 ng/μL in Hepes 35 mM, LiCl 5 mM pH 7.4 was incubated with Parvulins at the final concentration of 50 ng/μL, then Chymotrypsin was added at a final concentration of 150 ng/μL. The fluorescence of EDANS was measured at 510 nm upon excitation at 340 nm in a range of time from 0 to 4 h. The procedure consisted of the following steps:

**1.** Dispensing 12.5 μl of buffer into 384-wells of black OptiPlate-384;

**2.** Addition of 12.5 μl of FRET substrate at 60 ng/μL;

**3.** Addition of 12.5 μl of recombinant Parvulins at 200 ng/μL;

**4.** 15 min incubation at room temperature in the dark;

**5.** Addition of 12.5 μl of Chymotrypsin solution at 600 ng/μL;

**6.** Reading fluorescence over time, (λex = 340 nm and λem = 510 nm).

Experiments were run in triplicate and reported as averaged values. The deviation from the mean (standard error) was calculated using the GraphPad Prism 5.0 software.

## Results

### Full-Length N-Terminal His6-Tagged Par17 and Pin1 are Efficiently Overexpressed in *E. coli* Cells

Recombinant full-length Par17 was efficiently overexpressed in BL21(DE3)pLysS *E. coli* cells as N-terminal His6-tagged protein using the commercial expression vector pET14b. Optimal expression conditions were identified by varying culture temperature, IPTG concentration and incubation times (for details see Materials and Methods). As detected by SDS-PAGE analysis the target protein was expressed under all conditions tested (Fig. S2). The best conditions for overexpression of the protein were: cell growth at 22 °C for 16 h after induction with 0.5 mM IPTG when cells reached 0.6 OD_600nm_ (Fig. S2, panel B, see Table S1).

**Fig. 1:**
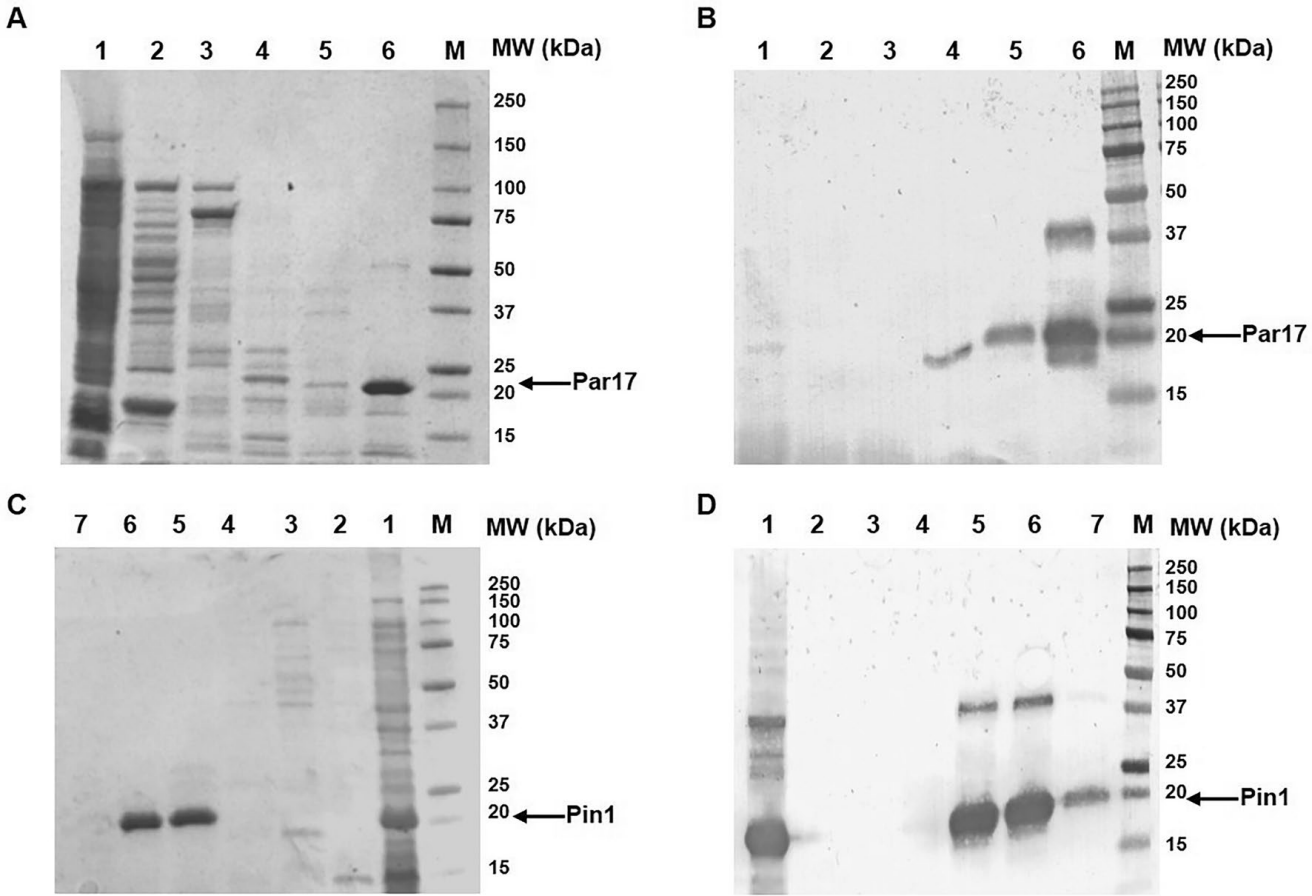
SDS-PAGE and WB analyses of Par17 (**A** and **B**) and Pin1 (**C** and **D**) fractions obtained during the purification. **A–B**: total extract fraction (lane 1), flow through fraction (lane 2), fraction eluted with imidazole 30 mM (lane 3), fraction eluted with imidazole 100 mM (lane 4), fraction eluted with imidazole 300 mM (lane 5), fraction eluted with imidazole 500 mM (lane 6). **C–D**: total extract fraction (lane 1), flow through fraction (lane 2), fraction eluted with imidazole 30 mM (lane 3), fraction eluted with imidazole 100 mM (lane 4), fraction eluted with imidazole 300 mM (lane 5), fraction eluted with imidazole 500 mM (two fractions, lane 6 and 7). M: protein standards

The protein was affinity purified on a Ni–NTA column from a colture of 600 ml. Elution was achieved through a step gradient of imidazole from 0 to 500 mM. SDS-PAGE and WB analysis of eluted fractions showed that Par17 was recovered starting from 100 mM imidazole; most protein was recovered in the fraction at 500 mM imidazole. The final product migrated as a single band of about 18 kDa, in accordance with the intact protein MW (Fig. 1A and B). However, WB analysis showed a second band of about 37 kDa in the more concentrated protein fractions, suggesting that the protein underwent dimerization in these conditions (Fig. 1B). After this single affinity chromatography step about 20 mg of pure protein per litre of colture were recovered.

The full-length Pin1 was also efficiently expressed in BL21(DE3) *E. coli* cells with a His6-tag at the N-terminus (Fig. S3 and Table S1), using the optimized expression protocol. The protocol is new compared to that reported in literature (see Materials and Methods for details and Table S1) [29, 30]. Starting from a colture of 600 ml, 13.2 mg of homogeneous protein (22 mg/L colture) were obtained after a single Ni–NTA affinity chromatography step. The resulting protein was estimated to be more than 95% pure by SDS-PAGE analysis and was detected as a band at about 18 kDa (Fig. 1 C) in accordance with the calculated MW. As observed with Par17 a second band at about 37 kDa compatible with the protein dimer, was detected by WB (Fig. 1D).

The identity of the purified proteins was confirmed by LC–MS analysis. The deconvoluted MS spectra showed for the final recombinant products MWs of 18,771.81 Da and 20,406.69 Da for Par17 and Pin1 respectively (Fig. S4A, B), which well-agree with the calculated MWs.

### Parvulins Catalytic Domains Were Overexpressed in *E. coli* Cells

The catalytic domains of Par17 and Pin1 were also expressed and purified as GST-fused polypeptides (hereafter, GST-Par17(60–156) and GST-Pin1(50–163), respectively) to compare their structural and functional properties with those of the full length proteins.

As reported in Fig. S5A and B, using the SOC medium, both domains were efficiently overexpressed in all conditions tested. For the large-scale expression we grew the cells at 22 °C for 16 h after induction with IPTG 1.0 mM when the OD_600nm_ reached 0.6 (Fig. S5A and B, lane 5). Similar results were obtained growing cells at 37 °C for 3 h after induction with IPTG 1.0 mM when the OD_600nm_ reached 0.6 (Fig. S5A and B, lane 3).

**Fig. 2:**
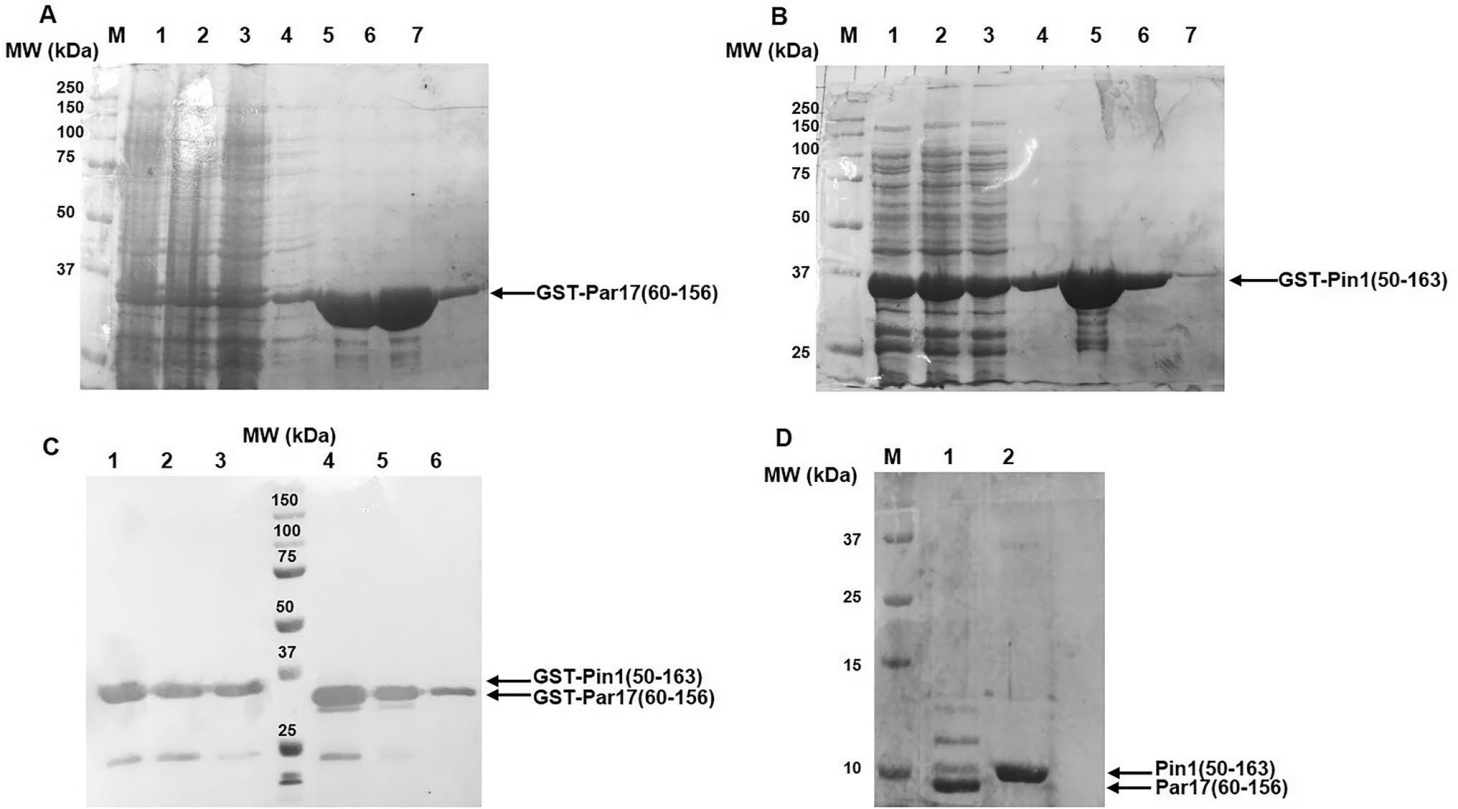
SDS-PAGE analyses of GST-Par17(60–156) (**A**) and GST-Pin1(50–163) (**B**) fractions obtained during the purifications. A-B: Total extract (lane 1), flow-through fraction (lane 2), washing fraction (lane 3), fraction of the elution step at 10 mM GSH (lanes 4–7). (**C**) WB analysis of purified GST-Pin1(50–163) (lines 1–3) and GST-Par17(60–156) (lines 4–6), respectively. (**D**) Par17(60–156) (lane 1) and Pin1(50–163) (lane 2) after thrombin treatment and subsequent purification step on GSTrap. M: protein size markers

GST-fused proteins were purified using a GSTrap column. Pure protein were eluted with 10 mM GSH and fractions were analysed by 4–20% precast SDS-PAGE and WB (Fig. 2). The pure proteins migrated as single bands of about 37 kDa, in agreement with their calculated MWs (about 40 kDa) (Fig. 2A–C).

The GST-fusion protein was subsequently removed by treatment with thrombin followed by a second step of affinity capture on GSTrap column to remove the GST (see Materials and Methods section for details). Final products migrated on SDS-PAGE as bands at about 10 kDa, in agreement with their MWs (Fig. 2D). For Par17(60–156) two bands at higher MWs were observed, likely accounting for SDS-stable different conformations of the domain. The identity of the purified Par17(60–156) and Pin1(50–163) were confirmed by LC–MS analysis (Fig. S6).

### Oligomerization Studies of Recombinant His6-tagged Par17

**Fig. 3:**
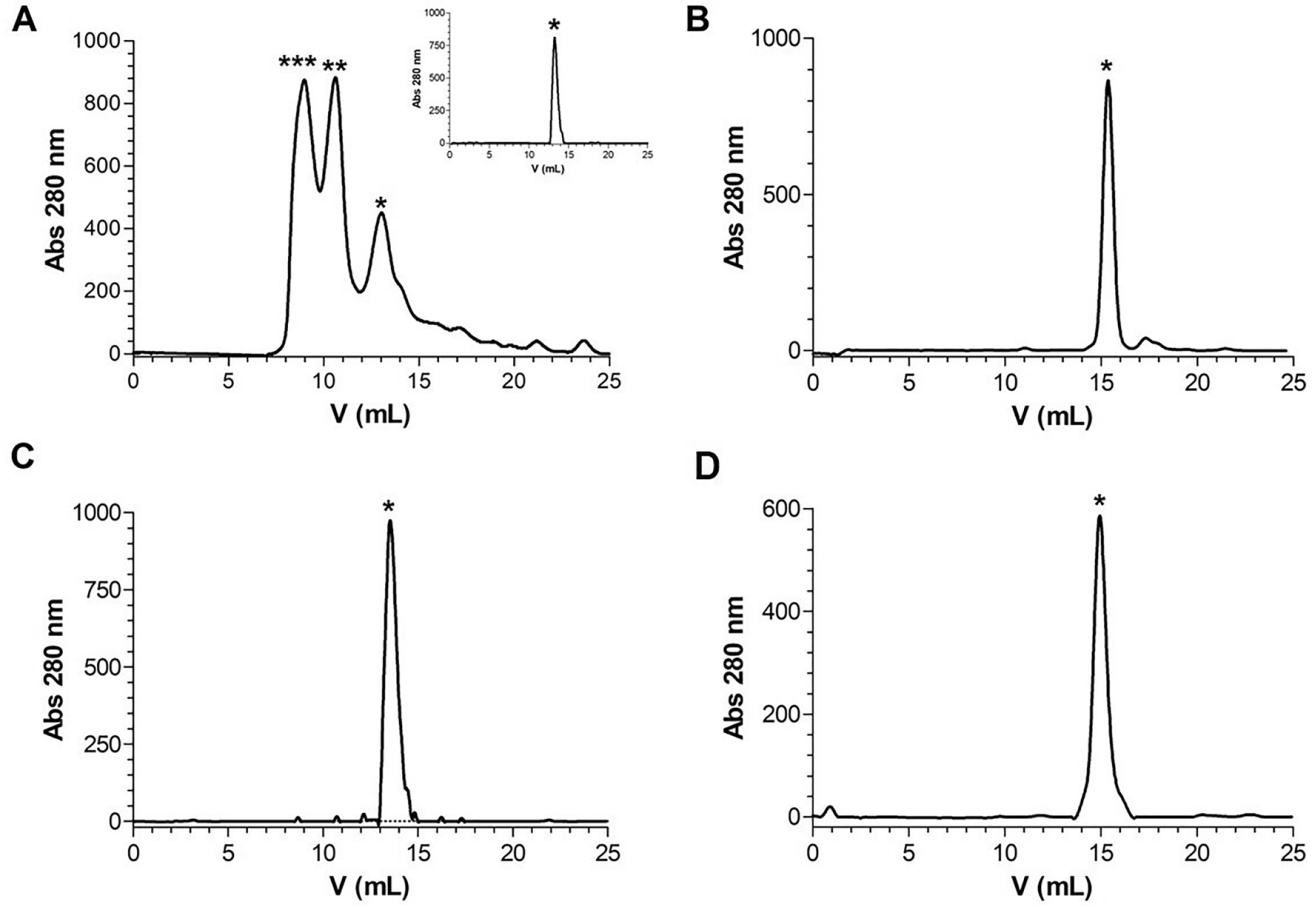
Representative SEC chromatograms of Par17 (**A**), Par17(60–156) (**B**), Pin1 (**C**) and Pin1(50–163) (**D**). SEC analysis of the isolated monomeric form of Par17 is reported in the inset graph of panel A. A single asterisk (*) indicates the elution volume expected for monomers, a double asterisk (**) indicates the elution volume expected for dimers/trimers and a triple asterisk (***) the one expected for oligomers

SDS-PAGE analyses of recombinant full-length Par17 and Pin1 have suggested that they are able to form oligomers/dimers stable in presence of SDS, reducing agents and high temperature. The oligomeric states of the proteins were therefore characterized by size exclusion chromatography. SEC chromatograms of Par17 showed three main peaks, the two highest having elution volumes of 9.1 and 10.2 ml respectively, while a third minor peak eluted at 13.3 ml (Fig. 3A). The molecular weight of each species was estimated using a standard calibration curve (see Material and Methods for details). As shown, MWs of about 70, 50 and 20 kDa were estimated, which could fit with a big oligomer, a trimer/dimer and a monomer, respectively.

To test these hypotheses, we next carried out SEC analyses on the catalytic domain Par17(60–156) lacking the N-terminal portion of the protein. In line with our first hypothesis, we observed a single species eluting at 15.4 ml, which is compatible with a monomer of about 11 kDa (Exper. 11 kDa; Estimated: 11.2 kDa) (Fig. 3B).

The same experiments performed on Pin1 and on its catalytic domain showed that both polypeptides were monomeric exhibiting a single peak at 13.5 and 15.0 ml, respectively (Fig. 3C and D). The estimated MWs were about 20 and 12 kDa for the full-length protein and the PPIase domain, respectively, in good agreement with those expected. The data thus strongly support the hypothesis that the oligomerization of Par17 is mediated by the extended N-terminus.

Interestingly, during the SEC separation, Par17 could be isolated in its monomeric form and was sufficiently stable to be detected in a SEC analysis and used in subsequent studies (see below and Fig. 3A, inset graph), even if aggregation reduced the recovery of the protein by more than 60%.

### The Monomeric Recombinant Par17 Is Folded in α-helix Rich Structures

**Fig. 4:**
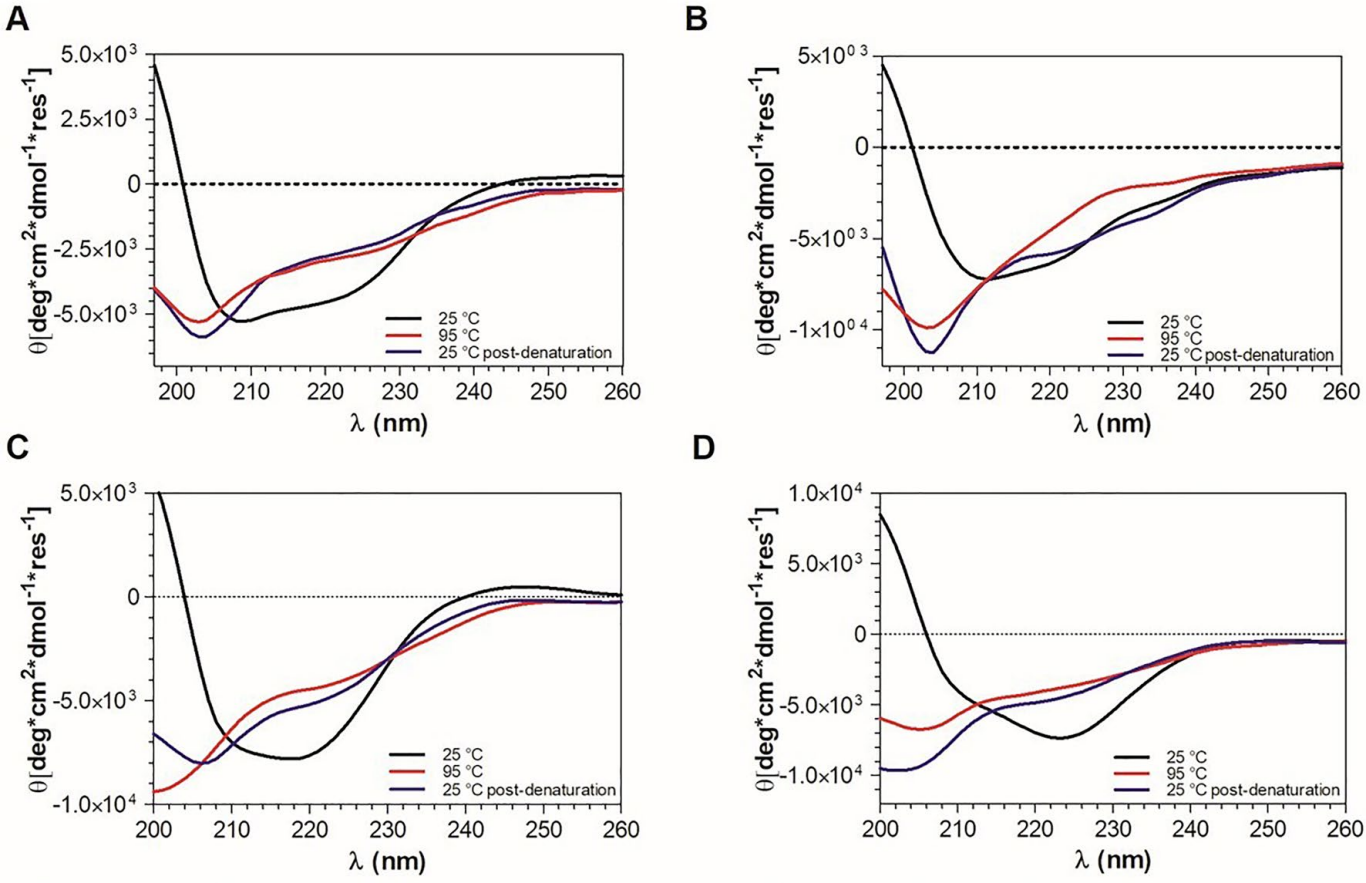
Overlay of the CD spectra of Par17 (**A**), Pin1 (**B**), Par17(60–156) (**C**) and Pin1(50–163) (**D**) recorded at 25 °C (black line), 95 °C (red line), and 25 °C after decreasing the temperature back to 20 °C (blue line, indicated in the graph as 25 °C post-denaturation)

The folding status of the proteins was comparatively assessed by far-UV CD spectroscopy. Full-length proteins were tested at 10 μM in 5 mM phosphate buffer at pH 7.4 and at 25 °C. As shown in Fig. 4A, the CD spectrum of Par17 was dominated by the presence of two minima at about 210 and 225 nm and one positive maximum at 195 nm, which are canonical hallmarks of mixed α-helical/β-strand structures (Fig. 4, black line). The relative quantitative analysis of the CD signal, performed using BestSel software [33], showed that the percentage of alpha-helix was about 40% and that the content of β-structure was about 14% (Table S2).

The stability to temperature of Par17 was evaluated by recording the CD signal upon heating. The protein exhibited a melting temperature (TM) of 55 °C (Fig. S7A), similar to that of very stable mesophilic proteins, and an isodichroic point at 204 nm, suggestive of a two-state transition from α-helical to unstructured conformations (Fig. S8A). In addition, the CD spectrum recorded at 95 °C showed that the protein retained residual ordinated conformations albeit it lost a significant level of structured regions, as suggested by the minimum at about 202 nm and the small shoulder at 220 nm (see Fig. 4A, red line). When the sample was cooled back from 95 °C to 25 °C, Par17 slightly recovered its folded structure indicating that the thermal denaturation was a partially reversible process. The spectrum indeed showed a red shift of the band at 202 nm (204–205 nm) and retention of the shoulder at 220 nm (Fig. 4A, blue line).

In similar conditions, the CD spectrum of Pin1 showed two minima (at ~ 210 and 222 nm) and one maximum (at 195 nm), featuring a well-folded α-helical structure (Fig. 4B, black line). Analysis of the spectrum with BestSel suggested that α-helices represented about 50% of all secondary structures (Table S2). The thermal denaturation analysis (Fig. S7B) showed that the protein has a T_M_ of 50 °C and that the conformational transition observed upon heating is characterized by an isodichroic point at about 206 nm, indicative of a two-state transition from structured to unstructured conformation (Fig. S8B). At 95 °C a significant alteration of the CD spectrum was observed (see Fig. 4B, red line). Indeed, an absolute minimum at 198 nm suggested that the protein was completely unfolded. Upon cooling at 25 °C the CD spectrum of the protein showed a strong minimum at 204 nm and a shoulder at 220 nm, suggestive of a slight recovery of the secondary structure.

The CD analysis of the catalytic domains of Par17 and Pin1 in the same buffer and at 25 °C showed that both proteins had positive maxima at 195 nm, negative minima at ~ 210 nm and strong negative bands at ~ 220–225 nm (Fig. 4C and D, black line). Compared to the full length proteins, the estimated content of α-helix dropped to 9.7 and 8.6% for Par17(60–156) and Pin1(50–163), respectively, compensated by a significant increase in the relative percentage of β- and unfolded regions (Table S2). The TM of the Par17 and Pin1 domains were 58 °C and 52 °C, respectively (Fig. S7 and S8 panels C, D), indicating that the catalytic domains are more or at least similarly stable compared to the corresponding full-length proteins. In addition, at 95 °C the catalytic domains completely unfolded, recovering only a minimal part of the original structure upon cooling back at 25 °C, indicating that the N-terminal regions serve the proteins to regain their structure following structural damages.

### Recombinant Par17 Is Endowed with Cis–Trans Prolyl Isomerase Activity

**Fig. 5:**
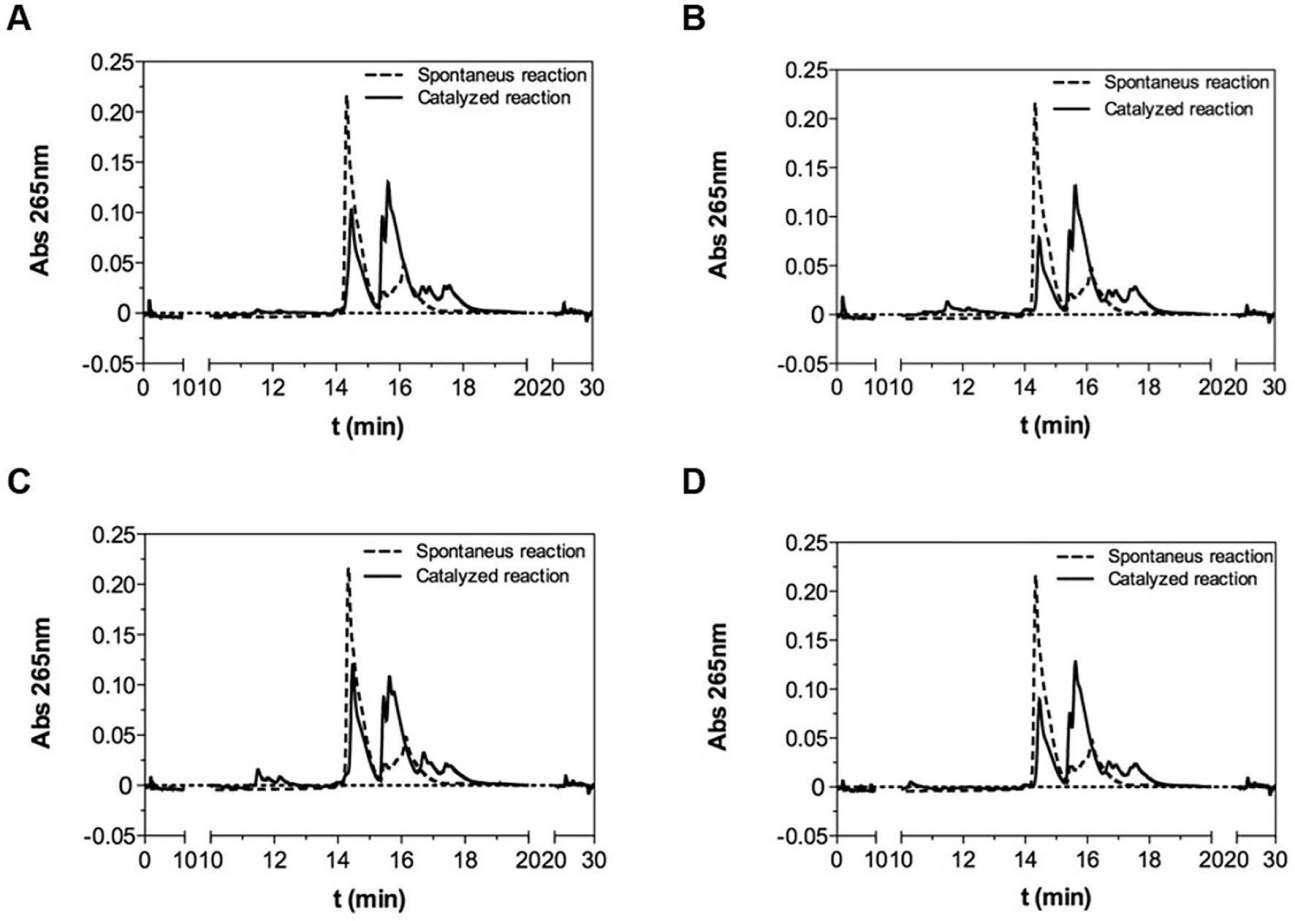
Chymotrypsin-induced proteolysis of the cis and trans isomers of the peptide substrate Fmoc-EAS(PO4)PRFAKA-NH_2_ as monitored by HPLC. Representative HPLC analyses of mixtures following spontaneous cis–trans isomerization (dashed lines) and after exposure (solid lines) to Par17 (**A**), Pin1 (**B**), Par17(60–156) (**C**) and Pin1(50–163) (**D**). Mixtures were, incubated 1 h at RT

We next assessed the catalytic activity of the purified proteins. To this aim we developed a chymotrypsin-based peptidyl-prolyl *cis–trans* isomerase assay specific for parvulins using a new peptide substrate containing the consensus sequence pSPRF [24] and a lysine (K) at the C-terminus to improve its solubility. Also, a glutamic acid (E) at the N-terminus was introduced to stabilize, through to the formation of a salt bridge with the K at the C-terminus, the putative *cis* conformation adopted by the X-P bond, as already reported in literature [32]. The final peptide substrate had therefore the sequence Fmoc-EApSPRFAKA-NH_2_ (pS = phospho-serine; theoretical MW = 1276.519 amu) and was synthesized on solid phase by the Fmoc strategy and characterized by LC–MS. Firstly, we tested whether the new substrate was processed by chymotrypsin independently of the presence of isomerases. Therefore, a mixture of substrate and chymotrypsin at a 1:2 molar ratio was analysed by HPLC at t0 and t1h (Fig. S9). At t1h a large amount of peptide resulted unprocessed by chymotrypsin (see peak at 14.60 min in Fig. S9) as detected by HPLC and LC–MS analyses (Fig. S9), showing that a substantial population of *cis* conformers of the peptide was present in our experimental conditions. Similar experiments were performed in presence of full-length Par17 and Pin1 and of their correspondent catalytic domains. In Fig. 5 the overlay of representative HPLC chromatograms of the various mixtures at t0 and t1h are reported. At t0 a single peak at a retention time (Rt) of about 14.60 min, corresponding to the intact peptide, was detected in all mixtures analysed. After 1 h a new peak with a Rt of about 15.90 min, corresponding to the chymotrypsin-cleaved peptide (Fig. 5), was detected in all mixtures (Fig. S10).

### Kinetic Characterization of Recombinant Parvulins

In order to develop and optimize a sensitive and robust FRET-Protease-Coupled peptidyl-prolyl *cis–trans* isomerase assay for assessing parvulins activity we also designed and prepared the peptide substrate Ac-EK(Dabcyl)pSPRFE(EDANS)KA-NH_2_ containing the Dabcyl/EDANS FRET pair [32]. The peptide was efficiently synthesized following a strategy previously reported [32], obtained with a high yield (~ 70%), and after purification was characterized by mass spectrometry.

**Fig. 6:**
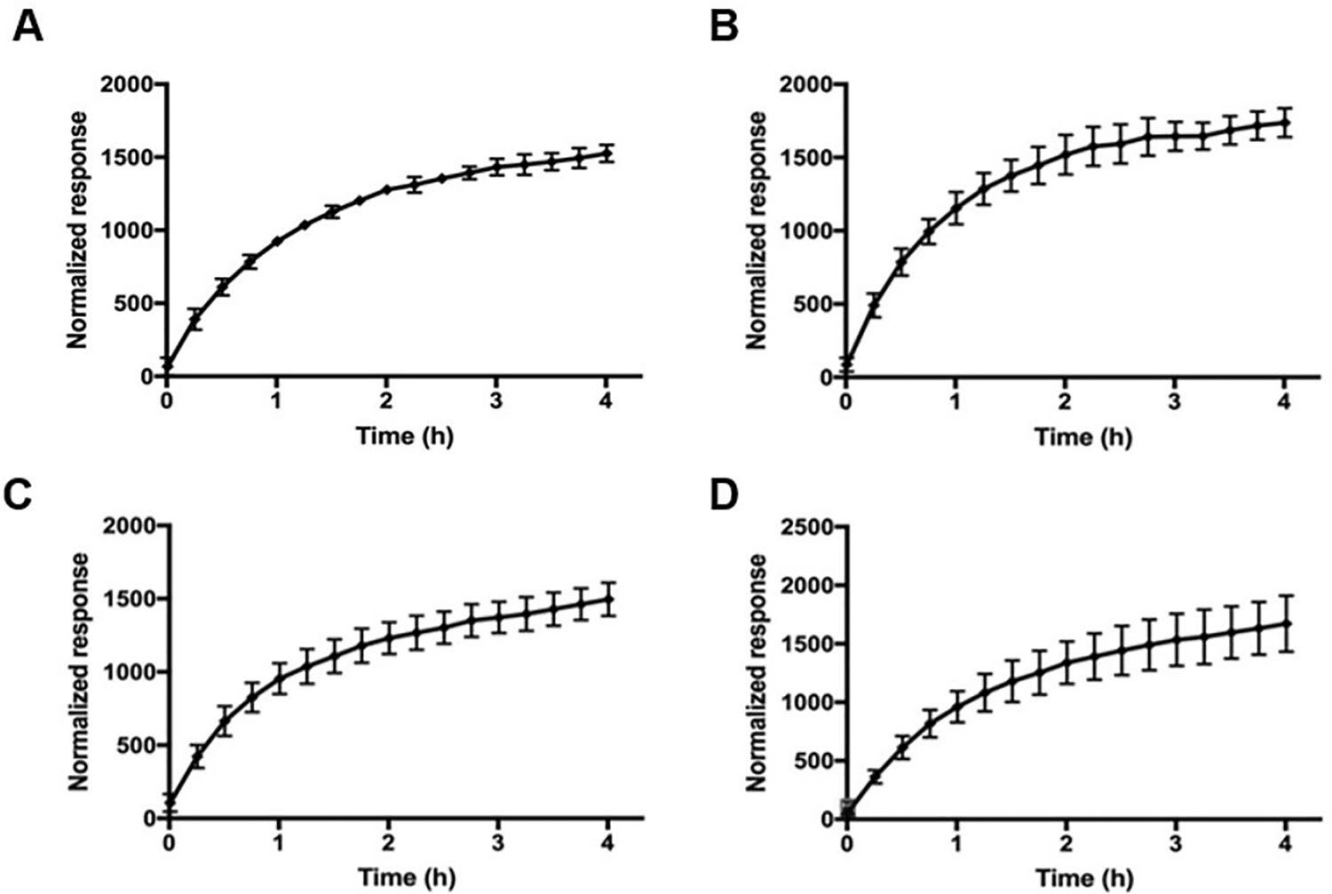
Time-dependent assays carried out using the FRET substrate at 15 ng/µl, chymotrypsin at 150 ng/µl and Par17 (**A**), Pin1 (**B**), Par17(60–156) (**C**) and Pin1(50–169) (**D**) at a final concentration of 50 ng/µl. Experiments were run in triplicate and reported as averaged values. The deviation from the mean (standard error) has been calculated with the GraphPad Prism 5.0 software

As shown in Fig. 6 incubation of recombinant Parvulins at room temperature with the substrate resulted in the specific enzymatic cleavage by chymotrypsin and a time-dependent increase of fluorescence intensity (λex/λem = 340 nm/510 nm). Values reported in Fig. 6, normalized respect to those obtained in the spontaneous reaction (substrate + chymotrypsin), are linearly related to the extent of Parvulins-mediated substrate isomerization. Values at 1 h reaction time were used to determine the Km and Kcat (Table 1).

**Table 1:** Value of Km, Vmax, Kcat and Kcat/Km calculated for each recombinant Parvulin tested

Entry	Km (M)	Vmax (M/s)	Kcat (s^−1^)	Kcat/Km (M^−1^/s)
Pin1	46,5*10^–6^	2.7*10^–6^	1112	2.4*10^7^
Par17	40,2*10^–6^	2.5*10^–6^	910.9	2.3*10^7^
Pin1(50–163)	48,2*10^–6^	2.7*10^–6^	744.8	1.5*10^7^
Par17(60–156)	62,5*10^–6^	3.1*10^–6^	708.5	1.1*10^7^

Experiments were run in triplicate and reported as averaged values. The deviation from the mean (standard error) has been calculated with the GraphPad Prism 5.0 software

Results show that all recombinant proteins have a peptidyl-prolyl *cis–trans* isomerase activity and that all have quite similar Km (Table 1). The Kcat are instead significantly lower for the two catalytic domains suggesting that while both proteins and domains efficiently recognize the substrate (similar Km) the catalytic activity is somehow improved in the full length proteins.

## Discussion

We report here the expression and purification protocols for the recombinant production of human Par17 (Par17) together with its structural and functional characterization. The protein has been efficiently expressed for the first time with a short tag (His6-tag) in *E. coli* cells and purified to homogeneity by a single step of affinity chromatography. The analysis of the oligomeric status of Par17 has revealed that the monomeric protein is in equilibrium with a large population of soluble oligomers, detectetable by both WB and SEC analysis, showing that the protein, in physiological conditions, may exist under various oligomeric forms. The monomeric form of the protein has been efficiently separated from the other oligomers through SEC and did not re-associate, indicating that it is stable enough to be isolated and characterized, although the final material recovered was only about 30 ÷ 40%. Since the catalytic domain of Par17 has no tendency to form oligomers in the same experimental conditions we hypothesize that oligomerization is mediated by the extended amphipathic N-terminal portion of the protein (Fig. S1). This hypothesis well fit with previous evidence which identified the amphypatic helices as inducer of oligomerization in addition to interacting with membrane lipids [34–36]. We also noticed that the homologous Pin1, which shares a high structural and functional similarity with Par17, appears as a monomer and its N-terminal region is far more hydrophilic compared to Par17 (Fig. S1). We thus hypothesize that the aggregation propensity of Par17 is also likely due to the various hydrophobic residues like leucines, methionines and valines, other than a stretch of glutamines known to promote self-association (Fig. S1), occurring in its N-terminal tail [37].

One isolated the monomeric recombinant Par17, like Pin1, adopts a mixed α-helix and anti-parallel β-strand arrangements, which well fit with the 3D structure reported for the catalytic domains of Pin1 and Par14/17 [20, 30]. Removal of the N-terminal part of Par17 and Pin1 significantly reduces the relative content of alpha-helix in favor of β-structures and unfolded regions, indicating that for both proteins the N-terminal region is crucial for the correct folding while the catalytic core, mostly composed by β-structures, is not much perturbed, in agreement with the retained enzymatic activity of the isolated catalytic domains (see below).

The ability of Par17 to catalyze the *cis–trans* interconversion has been assessed using in vitro assays. To this aim a FRET-based homogeneous assay amenable to HTS campaigns has also been developed. In particular, we have designed and prepared a new FRET substrate that is efficiently recognized and processed by both Parvulins in about 1 h, a time short enough for automated HTS methods. Notably, the results obtained for Pin1 and its catalytic site are in perfect agreement with previous results, further validating the new assay [38]. The assay has shown that the recombinant Par17 exhibits a *cis–trans* prolyl-peptidyl isomerase activity roughly similar to that of full-length Pin1. Moreover, no huge differences of catalytic activity have been observed between the full-length protein and the catalytic domain, indicating that the N-terminus does not play a role in the PPIase activity, at least in our simplified system.

Altogether, the data here presented may be helpful for subsequent studies aimed at the structural and functional characterization of Par17.

## Supplementary Information

Below is the link to the electronic supplementary material.

Supplementary file1 (DOC 3403 KB)
