# Supplementary material for: Expression, Purification, Structural and Functional Characterization of Recombinant Human Parvulin 17
Source: Mol Biotechnol. 2022 Apr 25;65(3):337–49. doi: 10.1007/s12033-022-00493-1 (PMC9935730; doi:10.1007/s12033-022-00493-1)
Supplement: Supplementary file 1 — Supplementary file1 (DOC 3403 KB) [file 12033_2022_493_MOESM1_ESM.doc]

**Supplementary Materials**

**Expression, purification, structural and functional characterization of recombinant human Parvulin 17.**

Par14 -----------------------------------MPPKGKSGSGKAGKGGAASG--SDS

Par17 -----**MPMAGLLKGLVRQLERFSVQQQAS**-----KMPPKGKSGSGKAGKGGAASG--SDS

Pin1 **MADEEKLPPGWEKRMSRSSGRVYYFNHITNASQWERPSG**NSSSGGKNGQ**GEPARVRCSHL**

Par14 ADKKAQGPK-GGG**NAVKVRHILCEKHGKIMEAMEKLKSG-MRFNEVAAQYSEDK-ARQGG**

Par17 ADKKAQGPK-GGG**NAVKVRHILCEKHGKIMEAMEKLKSG-MRFNEVAAQYSEDK-ARQGG**

Pin1 **LVKHSQSRRPSSWRQEKITRTKEEALELINGYIQKIKSGEEDFESLASQFSDCSSAKARG**

Par14 **DLGWMTRGSMVGPFQEAAFALPVSGMDKPVFTDPPVKTKFGYHIIMVEGRK**

Par17 **DLGWMTRGSMVGPFQEAAFALPVSGMDKPVFTDPPVKTKFGYHIIMVEGRK**

Pin1 **DLGAFSRGQMQKPFEDASFALRTGEMSGPVFTDS------GIHIILRTE--**

**Fig. S1: Sequence alignment of human parvulins.** Pin1 has two domains connected by a flexible linker. The N-terminal domain called “WW” (referring to two invariant Trp residues), spanning residues 1-39 (shown in blue), which targets the enzyme to pSer/Thr-Pro motifs in substrates, and the C-terminal PPIase domain (residues 50-163, shown in red) [1]. Par14 has a N-terminal domain, spanning residues 1-35 (shown in black) and the PPIase domain, spanning residues 36-131 (red). Compared to Par14, Par17 has an additional fragment of 25 residues at the N-terminus (orange). The catalytic domain spans residues 60-156 (red) [2].

**
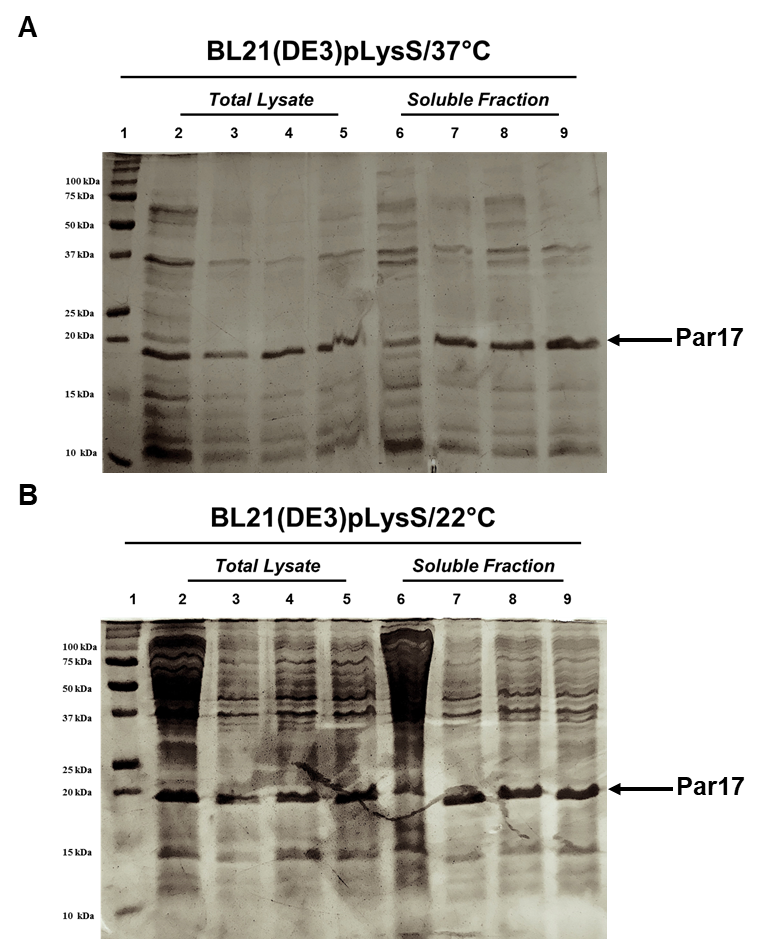
**

**Fig. S2: Recombinant expression and solubility of N-terminal His6-tagged Par17 in *E.coli* cells** (**A**, **B**) Coomassie-stained 15% SDS-PAGE gel showing total (from lane 1 to 5) and soluble fractions (from lane 6 to 9) from BL21(DE3)pLysS *E. coli* cells expressing full-length Par17 protein, cultured in SOC medium with different concentration of IPTG, and growing up after induction either at 37 °C for 3 h (panel A) or 22 °C for 16 h (panel B). For both panels is shown the protein size standard (line 1); not induced cells (lines 2 and 6); induced cells with IPTG at 0.3 mM (lines 3 and 7), with IPTG 0.5 mM (lines 4 and 8) and with IPTG 1.0 mM (lines 5 and 9). An intense band with a molecular weight (MW) of 18 kDa, compatible with the MW of Par17 full-length protein is observed in all conditions tested.

**Pin1**

**Pin1**

**A**

**B**


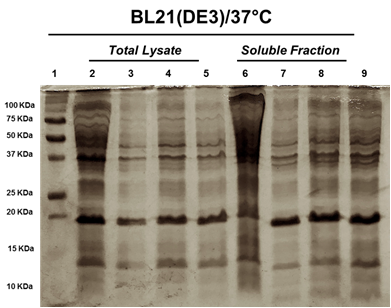

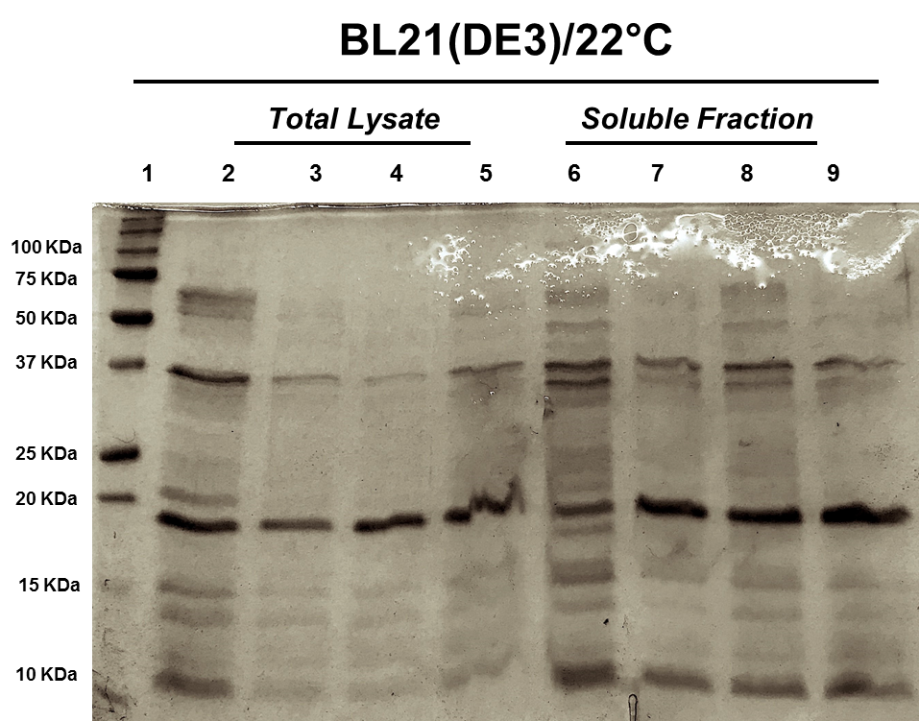


**Fig. S3: Recombinant expression of N-terminal His6-tagged Pin1 in *E. coli*. (A, B)** Coomassie-stained 15 % SDS-PAGE gel showing total (from lane 1 to 5) and soluble fractions (from lane 6 to 9) from BL21(DE3) *E. coli* cells expressing full-length Pin1 protein, cultured in LB medium with different concentration of IPTG, and growing up after induction either at 37 °C for 3 h (panel A) or 22 °C for 16 h (panel B). For both panels is shown the protein size standard (line 1); not induced cells (lines 2 and 6); cells induced with IPTG at 0.3 mM (lines 3 and 7), with IPTG 0.5 mM (lines 4 and 8) and with IPTG 1.0 mM (lines 5 and 9). An intense band with a molecular weight (MW) of 18 kDa, compatible with the MW of Pin1 full-length protein is observed in all conditions tested.


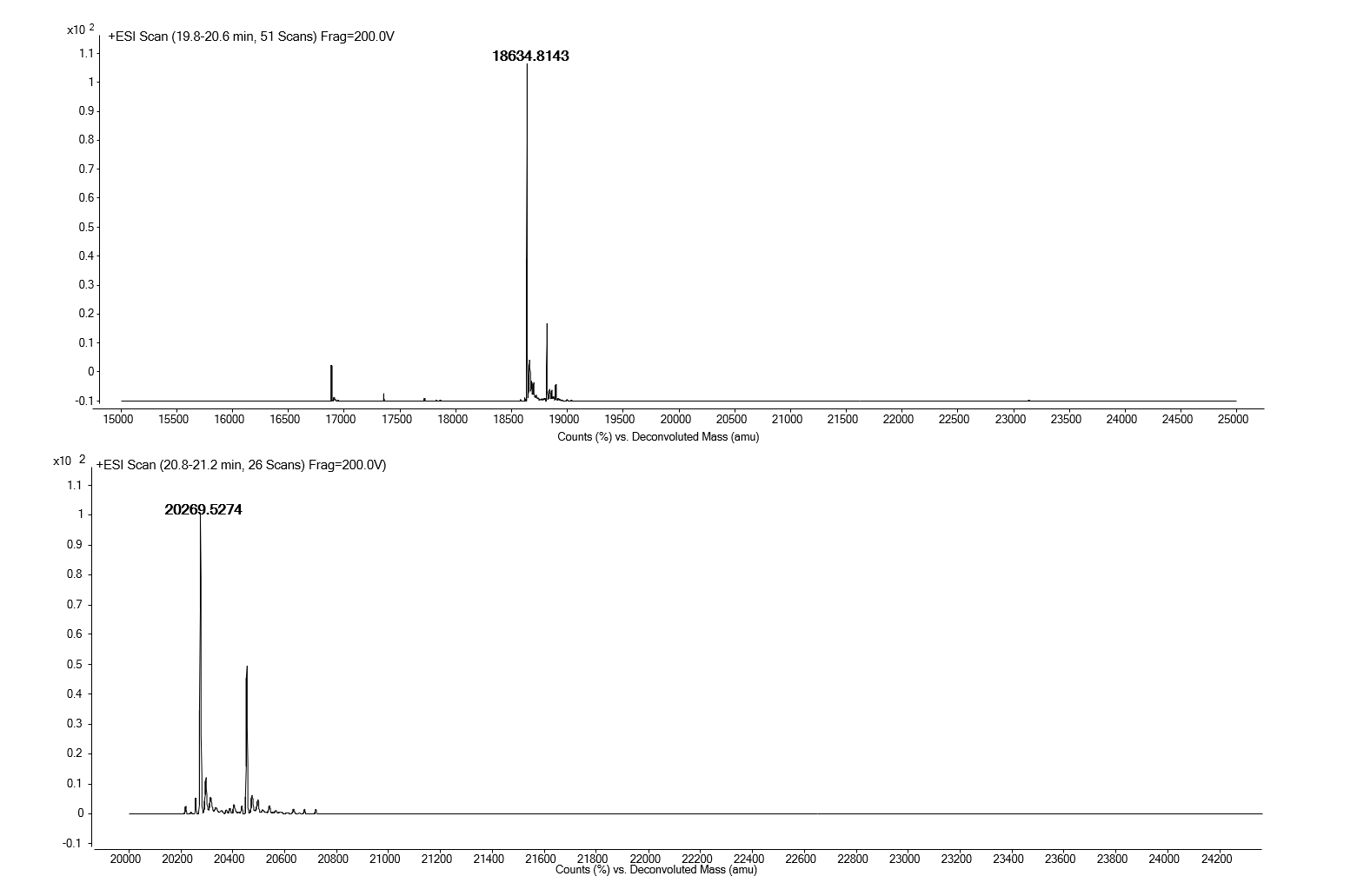


**Fig. S4:** Deconvolution of experimental mass spectrum with average mass peaks labelled of Par17 and Pin1 full-length proteins (upper and lower panels, respectively).


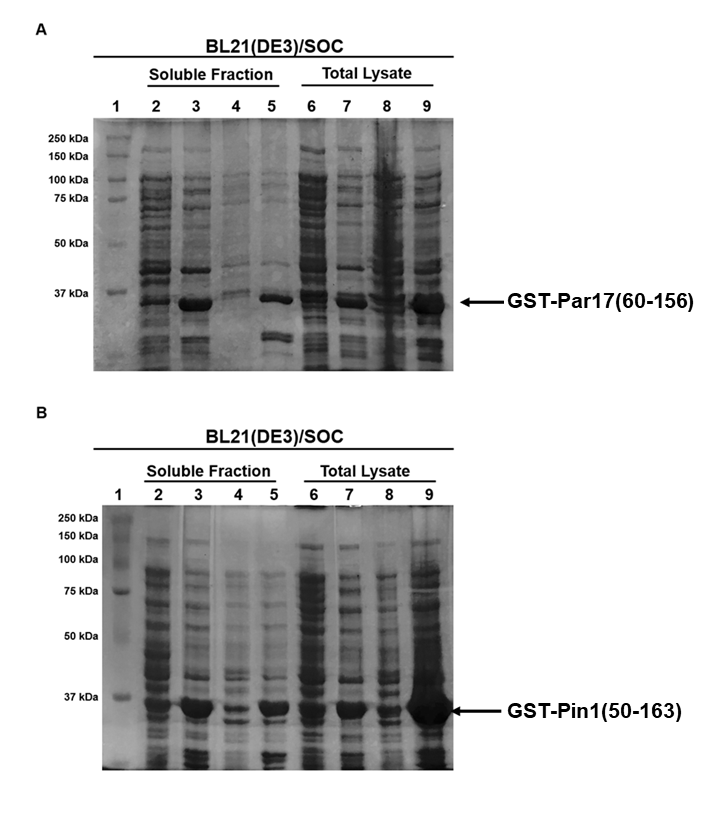


**Fig. S5: Recombinant expression of N-terminal** **GST-fused Par17(60-156) and Pin1(50-163) in *E.coli cells*. (A, B)** Coomassie-stained 15 % SDS-PAGE gel showing total (from lane 1 to 4) and soluble fractions (from lane 5 to 8) from BL21(DE3) *E. coli* cells expressing GST-Par17(60-156) and GST-Pin1(50-163), cultured in SOC medium with different concentration of IPTG, and growing up after induction either at 37 °C for 3 h or 22 °C for 16 h. For both panels is shown the protein size standard (line 1); soluble fraction and total lysate of not induced cells (lines 2 and 6, respectively); soluble fraction and total lysate of cells induced with IPTG at 0.3 mM at 37°C for 3 h (lines 3 and 7, respectively), with IPTG 0.5 mM at 37°C for 3 h (lines 4 for the soluble fraction and 8 for the total lysate) and with IPTG 1.0 mM at 22°C for 16 h (lines 5 for the soluble fraction and 9 for the total lysate). An intense band with a molecular weight (MW) of 18 kDa, compatible with the MW of Pin1 full-length protein is observed in all conditions tested.


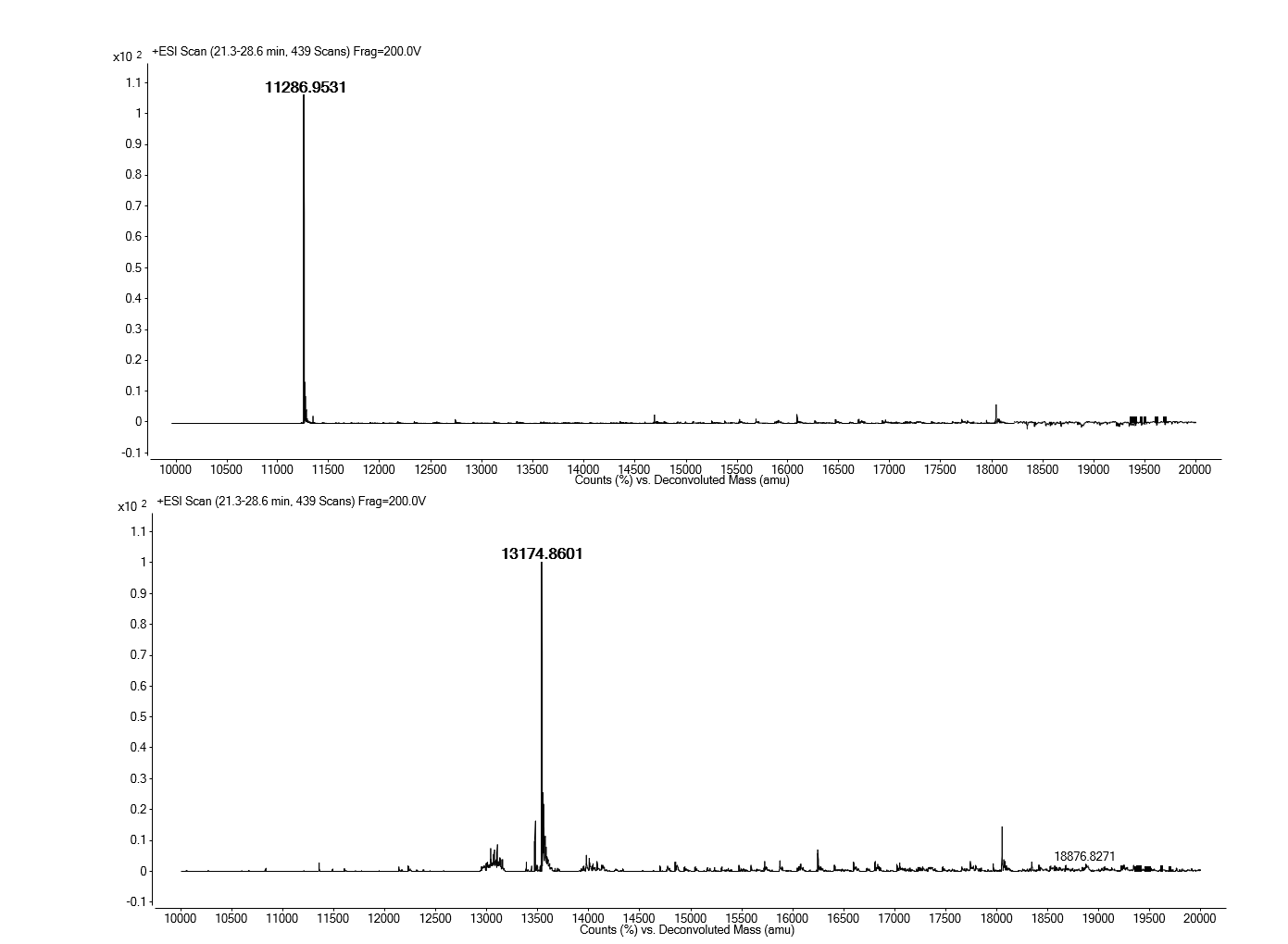


**Fig. S6:** Deconvolution of experimental mass spectrum with average mass peaks labelled of Par17(60-156) and Pin1(50-163) (upper and lower panels, respectively).


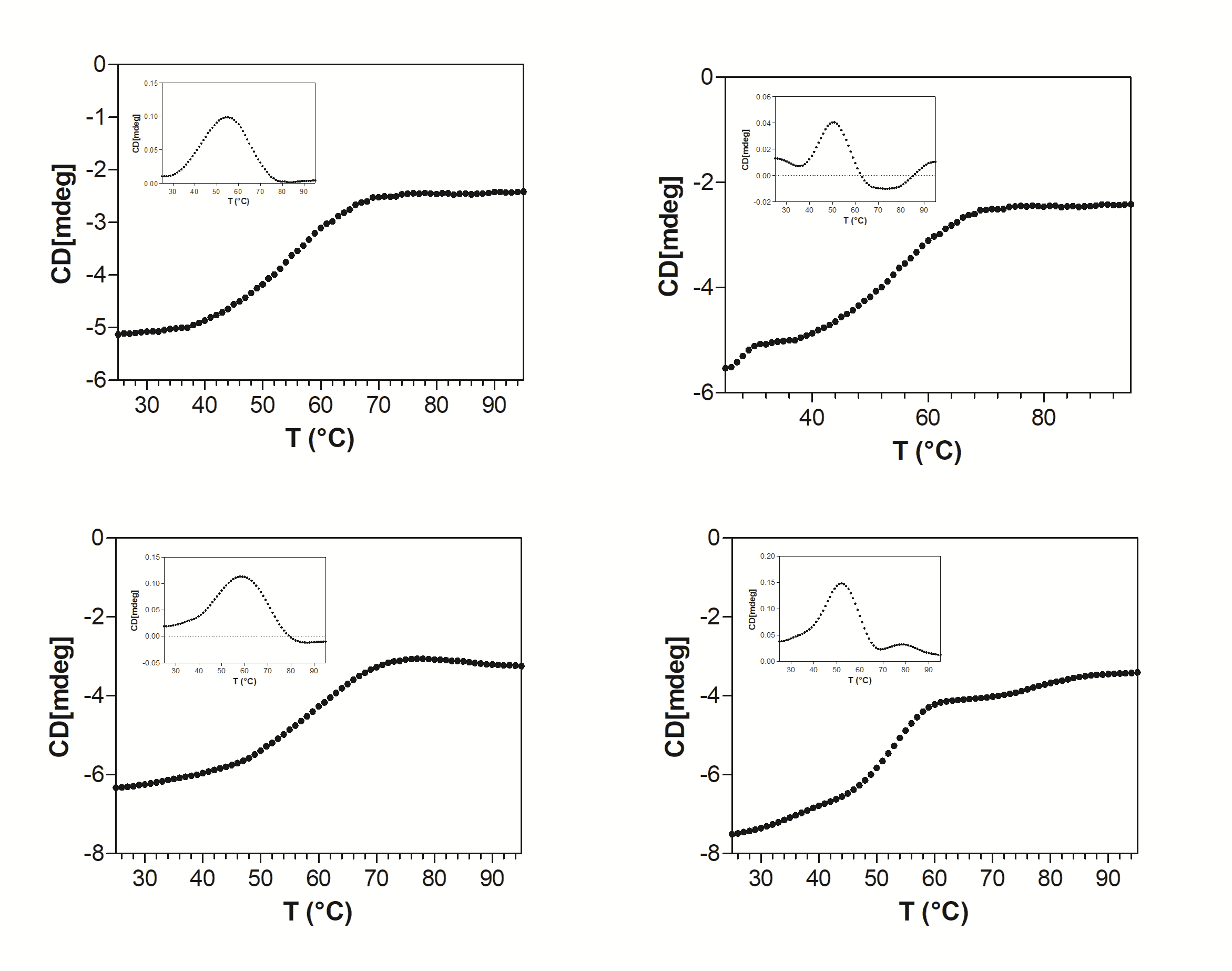


**A**

**B**

**C**

**D**

**Fig. S7:** Thermal denaturation profiles of Par17 (A), Pin1 (B), Par17(60-156) (C) and Pin1(50-163) (D). Inside the graphs the derivative plots were reported.


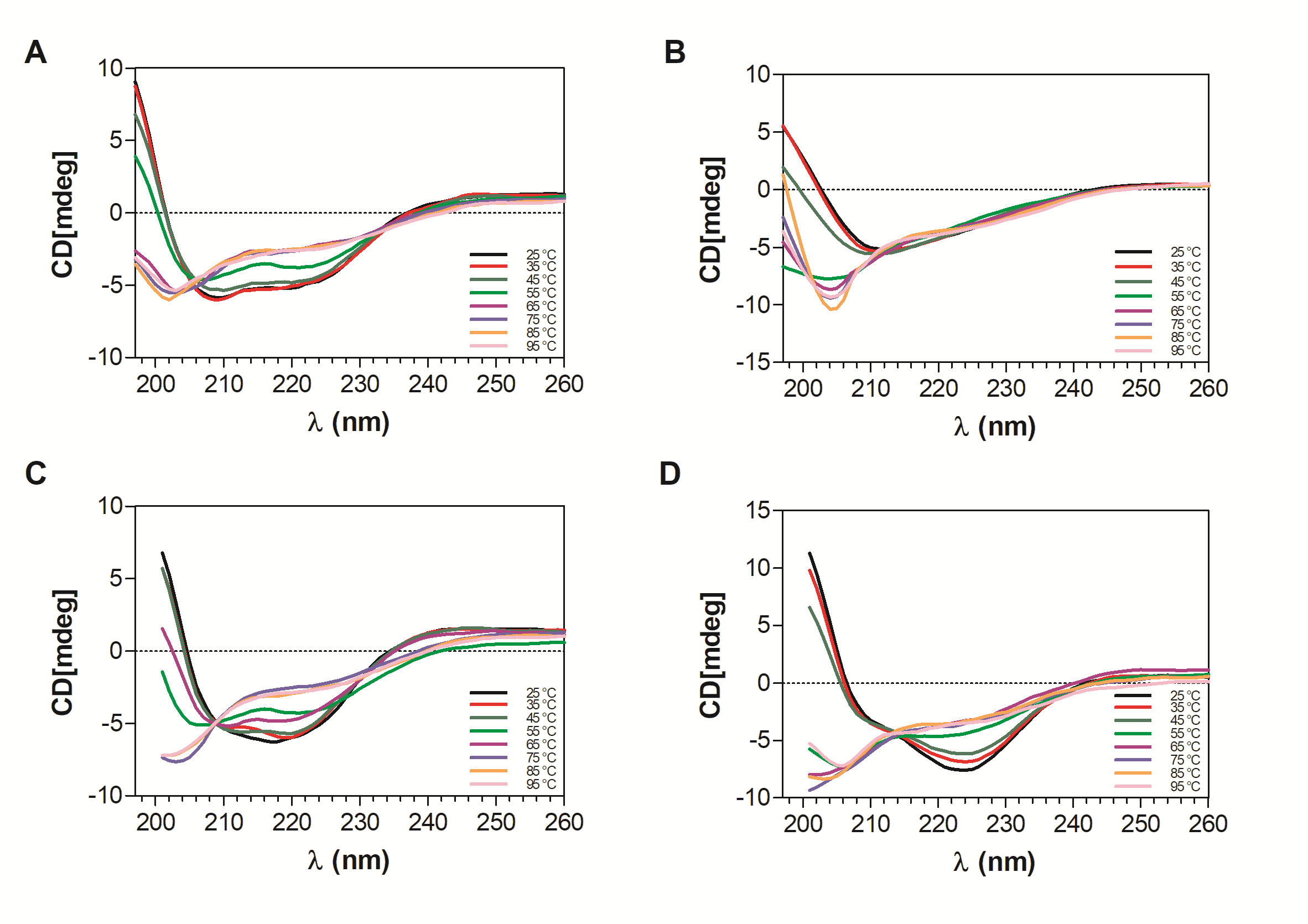


**Fig. S8:** Far-UV CD spectra of Par17 (A), Pin1 (B), Par17(60-156) (C) and Pin1(50-163) (D) collected at increasing temperatures from 25°C to 95°C.

**Fig. S9:** Overlay of HPLC traces of the mixture peptide substrate and chymotrypsin at t0 (black line) and at t1h (red line). The zoom of MS spectrum of the full-length peptide was also reported.


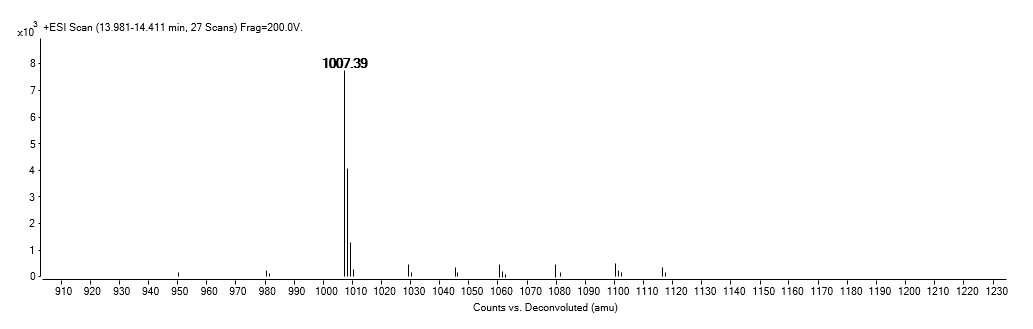


**Fig. S10:** Representative deconvolute MS spectrum of chymotrypsin-processed substrate in the peptidyl prolyl *cis-trans* assays in presence of Parvulins tested.

**Table S1. Optimal conditions for Parvulins overexpression in E. coli cells.**

| **Protein** | **E. coli strains** | **Cell media** | **Antibiotics** | **IPTG concentration**  **(mM)** | **Culture temperature after induction (°C)** | **Incubation time after induction (hour)** |
| --- | --- | --- | --- | --- | --- | --- |
| **Par17** | BL21(DE3)pLysS | SOC* | Ampicillin | 0.5 | 22 | 16 |
| **Pin1** | BL21(DE3) | LB** | Ampicillin | 1 | 37 | 3 |
| **Par17(60-156)** | BL21(DE3) | SOC* | Ampicillin | 1 | 22 | 16 |
| **Pin1(50-163)** | BL21(DE3) | SOC* | Ampicillin | 1 | 22 | 16 |

***SOC composition:** 2% tryptone, 0.5% yeast extract, 10 mM NaCl, 2.5 mM KCl, 10 mM MgCl2, 10 mM MgSO4, and 20 mM glucose, pH 7.0.

****LB composition:** 1% tryptone, 0.5% yeast extract, 1% NaCl (% m (g)/v).

**Table S2**: Quantification of secondary structure elements of full-length Parvulins and their catalytic sites by BestSel software [3].

|  | **ENTRY** | | | |
| --- | --- | --- | --- | --- |
| **Secondary structure element** | **Par17** | **Pin1** | **Par17(60-156)** | **Pin1(50-163)** |
| **Helix** | 40.3 % | 49.3 % | 9.7 % | 8.6 % |
| **Antiparallel β** | 13.7 % | 6.8 % | 31.4 % | 24.7 % |
| **Parallel β** | 0 | 0 | 0 | 10.9 % |
| **Turn** | 7.7 % | 6.4 % | 18.9 % | 13.1 % |
| **Others**  (loops/irregular, 3,10-helix, π-helix, β-bridge, bend) | 38.3 % | 37.5 % | 39.9 % | 42.7 % |

**References**

1. Ranganathan R., K.P. Lu, Hunter T., Noe J.P. (1997). Structural and Functional Analysis of the Mitotic Rotamase Pin1 Suggests Substrate Recognition Is Phosphorylation Dependent. Cell, 89(6): 875–886.
2. Mueller, J.W., Link, N.M., Matena, A., Hoppstock, L., Ruppel, A., Bayer, P. & Blankenfeldt, W. (2011). Crystallographic proof for an extended hydrogen-bonding network in small prolyl isomerases. J Am Chem Soc, 133, 20096-9.
3. Micsonai A., Wien F., Bulyáki É., Kun J., Moussong É., Lee Y-H, Goto Y., Réfrégiers M., Kardos J. (2018). BeStSel: a web server for accurate protein secondary structure prediction and fold recognition from the circular dichroism spectra. Nucleic Acids Res, 46(W1): W315-W322.
